# COVID-19: IgG seroconversion under intensive glucocorticoid treatment in a high-risk patient with minimal change disease

**DOI:** 10.1007/s00508-020-01776-w

**Published:** 2020-12-08

**Authors:** Michael Eder, Robert Strassl, Johannes Kläger, Christof Aigner, Florian Thalhammer, Željko Kikić

**Affiliations:** 1 Department of Internal Medicine III, Division of Nephrology and Dialysis, Medical University of Vienna, Vienna, Austria; 2 Division of Clinical Virology, Department of Laboratory Medicine, Medical University of Vienna, Vienna, Austria; 3 Department of Pathology, Medical University of Vienna, Vienna, Austria; 4 Department of Internal Medicine I, Division of Infectious Diseases and Tropical Medicine, Medical University of Vienna, Vienna, Austria

**Keywords:** Coronavirus, Hyperinflammatory syndrome, Kidney disease, Glomerulonephritis, Immunosuppression

## Abstract

In this case report we present a rare case of a patient with multiple risk factors for severe coronavirus disease (COVID 19) in whom intensive glucocorticoid treatment due to incipient nephrotic syndrome coincided with SARS-CoV‑2 infection. Despite this high baseline risk profile and the use of glucocorticoids the patient developed only mild disease including IgG SARS-CoV‑2 seroconversion.

The role of immunosuppressive strategies in patients with coronavirus disease (COVID-19) is controversially discussed in the literature [1, 2]. The World Health Organization (WHO) argues against the routine use of glucocorticoids due to prolonged viral shedding and reduced anti-viral type 1 interferon immune responses [3]. As suggested by Mehta et al. these concerns are countered by increasing evidence of COVID-19 subpopulations in which development of severe ARDS and multiorgan failure may be driven by virally induced secondary hyperinflammatory syndrome (SHS) and cytokine storm [1, 4]. Anecdotal reports in subjects without renal disease have suggested successful reversion of critical cases by non-selective (glucocorticoids, Intravenous immunoglobulin) or selective immunosuppression, i.e. interleukin 6 (IL-6) antagonism, mostly used as bail-out procedures. Prevention of severe COVID-19 and/or SHS at earlier disease stages through immunosuppression, however, may also be beneficial. So far, the evidence for prophylactic glucocorticoids treatment in nonsevere cases is limited by various uncertainties regarding optimal case selection, timing and dose of immunosuppression. Moreover, knowledge about immunocompromised patients with renal disease is derived mainly from case reports of renal transplant patients with varying clinical course but a not unsubstantial number of patients needed treatment at ICU units [5]. In this report we discuss a case of a patient with multiple risk factors for severe COVID-19 (obesity, renal disease, hypertension) in whom intensive glucocorticoid treatment due to incipient nephrotic syndrome coincided with SARS-CoV‑2 infection. Despite this high baseline risk profile and the use of glucocorticoids the patient developed only mild disease including IgG SARS-CoV‑2 seroconversion.

A 32-year-old male patient presented at our outpatient clinic with progressive fatigue, weight gain and edema. Physical examination showed an obese patient (163 kg) with lower extremity edema and mild hypertension (140/90 mm Hg). Laboratory findings (Table 1) confirmed a severe nephrotic syndrome (protein/creatinine ratio 6348 mg/g, hypoalbuminemia of 17 g/L), hypogammaglobulinemia (IgG 444 mg/dL) and no autoantibodies. The remaining laboratory findings (Table 1) showed normal creatinine and electrolytes or blood counts. Sonography revealed normal renal morphology. Renal biopsy showed no relevant pathologies in light microscopy and immunohistochemistry. Electron microscope findings confirmed the renal diagnosis of minimal change disease with podocyte flattening of 90%. After confirmation of the diagnosis high-dose glucocorticoid treatment with 75 mg aprednislone/day was established on 11 March due to progressive proteinuria despite use of ACE inhibitors (ramipril 1.25 mg q.d.). Furosemide 40 mg b.i.d was continued until remission. The patient developed afebrile mild respiratory infection symptoms (dry cough) 2 days later, diarrhea for 4 days and was tested positive for SARS-CoV‑2 (PCR) on 21 March. Patient history revealed category I contact on 10 March to an infected co-worker. No further symptoms occurred and the dose of glucocorticoids remained unchanged in the course of the quarantine of 14 days. A clinical and laboratory follow-up on 15 April showed complete remission of the nephrotic syndrome (Table 1) and virological clearance (negative nasopharyngeal and stool PCR; Roche Diagnostics, Mannheim, Germany) including IgG and IgA seroconversion (Euroimmun, Lübeck, Germany). SARS-CoV‑2 IgG and IgA was considered positive according to the manufacturer’s manual with an antibody ratio >1.1.

**Table 1:** Laboratory findings of the described case, obtained at the nephrology outpatient clinic

	13 February 2020	11 March 2020	15 April 2020
Hemoglobin, g/dl	13.9	14.5	16.4
Leukocytes, G/L	10.79	9.05	18.07
Thrombocytes, G/L	380	337	298
Serum creatinine, mg/dl	0.68	0.71	0.81
Albumin, g/L	17.0	16.2	47.1
LDH, U/L	277	227	243
CRP, mg/dl	0.26	0.29	0.04
P/C ratio in spot urine, mg/g	6384	9932	<0.05
A/C ratio in spot urine, mg/g	4391^a^	6510^b^	<0.05

*A/C* albumin/creatinine ratio, *LDH* lactate dehydrogenase, *P/C* protein/creatinine, *CRP* C‑reactive protein

^a^measured on 17 February

^b^measured on 6 March

This case delivers new insight into this patient group otherwise classified as high-risk and also illustrates that intensive glucocorticoid treatment did not negatively influence the course of disease in selected COVID-19 cases with native kidney disease.
